# 2333. Knowledge, attitudes, and practices regarding vaccination against COVID-19 among medical students at a private university in Lima – Peru

**DOI:** 10.1093/ofid/ofad500.1955

**Published:** 2023-11-27

**Authors:** Benjamin Ramos-Rojas, Sebastian Lopez-Rivera, Karla Tafur-Bances, Jose A Gonzales-Zamora, Jorge Alave

**Affiliations:** Universidad Peruana Cayetano Heredia, Lima, Lima, Peru; Universidad Peruana Cayetano Heredia, Lima, Lima, Peru; Universidad Peruana Cayetano Heredia, Lima, Lima, Peru; Infectious Disease Division. University of Miami, Miller School of Medicine., Miami, Florida; Universidad Peruana Union, Lima, Lima, Peru

## Abstract

**Background:**

Vaccination against COVID-19 is one of the most important measures to reduce the spread, morbidity, and mortality of the disease.

**Methods:**

We conducted a cross-sectional study to evaluate knowledge, attitudes, and practices regarding vaccination against COVID-19 in medical students at Universidad Peruana Cayetano Heredia (UPCH), for which an online survey was administered through e-mail and WhatsApp in July 2022. The following independent variables were evaluated: To be in the clinical phase of medical school (last four years), having more online classes ( >= 4 semesters with non-face-to-face classes), having received >= 3 doses of COVID-19 vaccine, and having reliable sources of information (scientific articles, conferences, and seminars). Through linear regression multivariate analysis, we assessed the factors associated with the number of correct answers (knowledge), the standardized attitude index (attitudes), and the number of vaccine doses received (practice).

**Results:**

A total of 352 medical students completed the survey. 96.6% of participants knew that vaccines improved immunity, and 91.2% believed they were effective in reducing hospitalizations, severe illness, and death. 98% of students agreed that the benefits of vaccination outweighed the risks, 90.9% would recommend the population to get vaccinated, and 59.1% agreed that vaccines were safe. 99.7% of respondents have received at least one dose of vaccine, of which 100% received at least two doses, 88% three, and 4.8% four or more. In the multivariate analysis, we found that being in the clinical phase of medical school was associated with a higher knowledge and a higher standardized attitude index. Having received >=3 doses was associated with a higher standardized attitude index. Lastly, having a higher standardized attitude index was associated with having received more vaccine doses. (Table. 1)

Table 1

**Figure:**
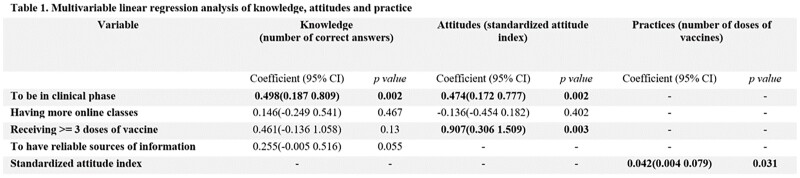

**Conclusion:**

Regarding vaccination against COVID-19, medical students at UPCH have a high level of knowledge, a positive attitude, and good practices. Our study suggests that educational strategies in the pre-clinical phase may be needed to improve knowledge and attitudes regarding vaccination. Also, a higher standardized attitude index may predict a higher number of vaccine doses.

**Disclosures:**

**All Authors**: No reported disclosures

